# Pharyngeal colonization and drug resistance profiles of *Morraxella catarrrhalis*, *Streptococcus pneumoniae*, *Staphylococcus aureus*, and *Haemophilus influenzae* among HIV infected children attending ART Clinic of Felegehiwot Referral Hospital, Ethiopia

**DOI:** 10.1371/journal.pone.0196722

**Published:** 2018-05-10

**Authors:** Wondemagegn Mulu, Endalew Yizengaw, Megbaru Alemu, Daniel Mekonnen, Derese Hailu, Kassaw Ketemaw, Bayeh Abera, Mulugeta Kibret

**Affiliations:** 1 Department of Medical Microbiology, Immunology and Parasitology, College of Medicine and Health Sciences, Bahir Dar University, Bahir Dar, Ethiopia; 2 AmharaPublic Health Institute, Bahir Dar, Ethiopia; 3 Department of Ear, Nose and Throat, College of Medicine and Health Sciences, Bahir Dar University, Ethiopia; 4 Department of Biology, Science College, Bahir Dar University, Bahir Dar, Ethiopia; Public Health England, UNITED KINGDOM

## Abstract

**Background:**

Asymptomatic pharyngeal colonization by potential bacteria is the primary reservoir for bacterial species within a population and is considered a prerequisite for development of major childhood diseases such as sinusitis, otitis media, pneumonia, bacteremia, and meningitis. However, there is dearth of data on the colonization and drug resistance pattern of the main bacterial pathogens in the pharynx of HIV infected children in Ethiopia. Therefore, this study determined the pharyngeal colonization and drug resistance profile of bacterial pathogens in HIV infected children attending ART clinic of Felegehiwot Referral Hospital (FHRH), Amhara Region, Ethiopia.

**Methods:**

A hospital based cross-sectional study was conducted from May 2016 to June 2017 at the ART clinic of FHRH. A total of 300 HIV infected children were enrolled in the study. Data on socio-demographic characteristics of the study participants were collected with face-to-face interview and patient—card review using structured questionnaire. Bacterial species were identified using standard bacteriological techniques. Drug susceptibility testing was performed using disk diffusion technique. Chi-square test was done to determine associations among variables.

**Results:**

The median age of the participants was 11 years. Overall, 153 (51%) of children were colonized by respiratory bacteria in their pharynx. Colonization rate was higher in children from mothers who had attained college and above levels of education than others (P = 0.04). It was also higher in children without the sign of malnutrition than others (P = 0.004). The colonization rate of *S*.*aureus*, *M*.*catarrhalis*, *S*.*pneumoniae* and *H*.*influenzae* were 88 (29%), 37 (12.3%), 31 (10.3%) and 6 (2%), respectively. *S*.*aureus*—*M*.*catarrhalis* concurrent colonization was found in 14 (4.7%) of children. Age (P = 0.03), schooling (P = 0.045) and history of running nose (P = 0.043) were significantly associated with *S*.*aureus* colonization. Living in urban setting (P = 0.042) and children from mothers with college and above levels of education (P = 0.002) were significantly associated with *M*.*catarrhalis* colonization. Majority of the isolates were resistant to penicillin (68.5%) and cotrimoxazole (52.5%).*S*.*aureus* isolates were resistant to penicillin (84.1%) and cotrimoxazole (51.1%).*M*.*catarrhalis* isolates were resistant to penicillin (94.6%), erythromycin (86.5%)and cotrimoxazole (78.4%). Overall, 99 (59.3%) of the isolates were multi-drug (MDR) resistant. The overall MDR rates among *S*.*aureus*, *M*.*catarrhalis* and *S*.*pneumoniae* isolates were 65.9%, 78.4% and 22.6%, respectively.

**Conclusions:**

Pharyngeal colonization of respiratory bacteria in HIV infected children is a major public health problem. Single and multiple antibiotic resistant is alarmingly high among respiratory colonizers. Therefore, regular screening of HIV infected children for culture and antimicrobial susceptibility testing is recommended to prevent the development of severe opportunistic infections.

## Introduction

Individuals infected with Human Immunodeficiency Virus (HIV) are at high risk of bacterial colonization and diseases [1, 2]. In the developing world, otitis media, pneumonia, meningitis and sepsis due to bacteria frequently occur in children with HIV [1, 2]. Upper respiratory tract (URT) colonization with pathogenic bacteria is a prerequisite for development of major childhood diseases such as bacterial sinusitis, otitis media, pneumonia, bacteremia and meningitis [1, 3, 4].

*Streptococcus pneumoniae* (*S*.*pneumoniae*), *Staphylococcus aureus* (*S*.*aureus*), *Haemophilus influenzae* (*H*.*influenzae*) and *Morraxella catarrrhalis* (*M*.*catarrhalis*) are the major asymptomatic colonizers of the nose and throat [1, 3, 4].*S*.*pneumoniae* is the leading cause of childhood bacterial pneumonia and it is responsible for at least800, 000 child deaths annually in developing countries [5, 6]. Each year, one million children die from pneumonia and invasive diseases [1, 2, 7, 8]. Globally, 4300 death occurs in children every day due to pneumonia. In Ethiopia, pneumonia is the leading infectious killer of children accounting for 28% of under five mortality [9–12].

Even with appropriate treatment, pneumococcal meningitis kills approximately one-third of affected African children. Nearly 30–60% of survivors develop long-term hearing loss, neurological defects, and neuropsychological impairment [2]. In sub-Saharan Africa, *S*.*pneumoniae* was found to account for >30% of meningitis cases in under 5 children, with a case fatality rate of > 50% [1].

*H*.*influenzae* is a major cause of ill health and premature death in infants and young children in developing countries [13]. *M*.*catarrrhalis* is also an opportunistic pathogen that causes pneumonia, sinusitis and otitis media in children and immunocompromised patients [13]. In persons infected with HIV, *S*.*aureus* infection account for significant morbidity [14, 15]. URT carriage is an important risk factor for staphylococcal septicemia. Carriers develop infection more frequently compared to non-carriers [15]. Otitis media is the most frequently reported childhood bacterial infection. *S*. *pneumoniae*, *H*. *influenzae* and *M*. *catarrrhalis* are the predominant documented etiologic agents of otitis media [5, 6].

Several studies stated that age, immune status, recent antibiotic use, crowding as occurs in day-care attendance, prior hospitalization, prisons, family size, number of siblings, poverty and smoking influence colonization of the human URT by bacterial pathogens [1, 3, 15].

The rapid spread of antimicrobial resistance among infectious organisms is a global public health problem [16]. Misuse of antibiotic provides selective pressure favoring the emergence of resistant strains not only in pathogenic bacteria, but also in the commensal flora of exposed individuals [16]. Carriage of penicillin and other antibiotic resistant *S*.*pneumoniae* in the pharynx of children is a worldwide emergence [1]. Moreover, the spread of methicillin resistant *S*. *aureus* (MRSA) in clinical as well as community settings became a real threat to public health [16].*M*.*catarrhalis* may also protect other respiratory pathogens from the action of penicillin or ampicillin by producing β-lactamase [17].

Though the pneumococcal conjugate vaccine is highly effective in reducing carriage and subsequent disease caused by *S*. *pneumoniae*, vaccination coverage is limited and difficult to prevent the spreading of non- vaccine serotypes [18]. Moreover, different reports demonstrated an inverse relationship between nasopharyngeal carriage of vaccine type *S*.*pneumoniae* and *S*.*aureus* [19] and increases the proportion of otitis media due to non-typeable *H*.*influenzae* following PCV7 vaccination [1, 20].

It is certain that the bacteria carried in the pharynx of HIV infected children reflect the infection-causing strains currently disseminating in the risk groups. Asymptomatic carriage is a prerequisite for developing invasive and noninvasive diseases, and carriers serve as sources of infection to others in the community and in the hospital. In Ethiopia, children are suffering from extremely high burdens of potential respiratory pathogenic diseases and therapy for such diseases remains empiric. However, to the best of our knowledge, colonization prevalence and drug resistance pattern by potential pharyngeal bacteria have not been studied in HIV infected children in Ethiopia. Therefore, the aim of this study was to determine the pharyngeal colonization and drug resistance pattern of respiratory bacteria in HIV infected children aged between 6–16 years attending the ART Clinic of Felegehiwot referral hospital (FHRH), Amhara National Regional State (ANRS), Ethiopia.

## Methods and materials

### Study design, period and setting

A hospital based cross-sectional study was conducted from May 2016 to June 2017 in anti-retroviral treatment (ART) clinic of FHRH, ANRS, and Ethiopia. FHRH is one of the government sponsored hospitals for ART services. The hospital provides both counseling and ART service to HIV infected patients. It has more than 273 beds offering different specialized services. Its ART clinic provides follow-up service to both pre-ART and ART pediatrics and adult patients by specialist and trained health professionals. The ART service is provided to HIV infected patients who are found in Bahir Dar and its vicinity. In addition, the hospital serves patients referred from different parts of the region for ART. The number of HIV positive children attending the ART clinic ranges from 1760–2200per year and this constitutes the source population.

### Sample size and sampling

The sample size was calculated using single population proportion formula (N = z2 p (1-p)/d2). It was determined by taking 5% degree of precision, 95% confidence level, a proportion of bacterial pharyngeal carriage, 33.3% [9]. Therefore, a sample size of 341 was calculated. However, complete data were obtained for only 300 HIV infected children. All HIV infected children attending ART clinic of FHRH were included conveniently until the required sample size was achieved.

### Variables

Pharyngeal colonization of respiratory bacteria such as *S*.*pneumoniae*, *S*.*aureus*, *M*.*catarrhalis* and *H*.*influenzae* were dependent variables while socio-demographic, clinical and other explanatory variables were independent variables.

### Data collection

Information on socio-demographic variables, schooling, habit of nose picking, habit of priming nail with teeth, living with health care workers, presence of younger siblings in the family and exposure to passive smoking were collected from each participant by face-to-face interview using a structured questionnaire. Moreover, clinical information such as the status of respiratory tract infection during the last month, sign of malnutrition, runny nose for the last two weeks, and coryza for the last two months was collected through patient card review [S1 Table].

### Pharyngeal sample collection and processing

After the completion of the interview, pharyngeal specimens were obtained by the same trained general practitioners working on ear, nose and throat clinic of FHRH according to standard microbiological techniques [7]. Two pharyngeal specimens per child were collected with a sterile synthetic cotton swab on flexible aluminum wire by rotating 4–5 times both clockwise and counterclockwise directions before withdrawal. After sampling, swabs were placed immediately on Amies transport medium (Oxoid, UK) and transported to the microbiology laboratory of Amhara Public Health Institute (APHI). One of the swab sample was inoculated on Manitol Salt Agar and the other swab was first inoculated to Sheep Blood Agar supplemented with 5 mg/ml Gentamicin plates (Oxoid, UK), and then Chocolate Agar within four hours of collection. Blood and Chocolate Agar plates were incubated in candle jar to generate about 5% CO_2_. All of the inoculated media were incubated at 37°C for 18–24 hrs.

### Identification of bacterial isolates

*S*.*pneumoniae* isolates were identified by alpha hemolytic colonies on blood agar which were susceptible to optochin and bile. *H*.*influenzae* was identified by colorless medium size colonies on Chocolate Agar and strict requirement for X (Hemin) and V (NAD+) factors. Similarly, *M*.*catarrhalis* isolates were identified by non- hemolytic grey to white colonies on Blood Agar which were catalase and oxidase test positive. Golden-yellow colonies on Mannitol Salt Agar which were oxidase and catalase test positive were considered as *S*.*aureus* [7].

### Antimicrobial susceptibility testing

Antimicrobial susceptibility testing was done on Mueller Hinton agar supplemented with 5% sheep’s blood (Oxoid, UK) by Kirby—Bauer disk diffusion method. The antimicrobial agents tested were: cefoxitin (30μg), clindamycin (30 μg), cotrimoxazole (25μg), ciprofloxacin (5 μg), erythromycin (15 μg), tetracycline (30μg), penicillin (10 IU) and chloramphenicol (30μg) (Oxoid, England) [21]. A 0.5 McFarland standard was used to standardized the turbidity of the inoculums suspension. Within 15 minutes after adjusting the turbidity of the inoculums suspension, a sterile cotton swab was dipped into the adjusted suspension. The dried surface plates were inoculated by streaking the swab over the entire sterile agar surface. The antimicrobial disks were placed on the lawn of bacterial isolates using sterile forceps. Inoculated media were incubated in a 5% CO_2_ atmosphere for 18–24 hours at 37°C. The diameter of zone of inhibition measured using caliper. The results were interpreted using the standard zone sizes of the Clinical and Laboratory Standard Institute (CLSI, 2015) guidelines [22]. All *S*.*aureus* isolates were subjected to cefoxitin disc diffusion test on Mueller Hinton Agar plates. Penicillin susceptibility for *S*.*pneumoniae* was determined using oxacillin disks as reagents for MIC determination were not available. Plates were incubated at 35°C for 18–24 hours and inhibition zone diameter of ≥ 21mm reported as methicillin resistant and ≥ 22 mm considered as methicillin sensitive [22].

### Quality control

Reference strains of *S*.*aureus* ATCC25923, *S*.*pneumoniae* ATCC49619 and *H*. *Influenzae* ATCC 49241were used for quality control for antimicrobial susceptibility testing. Detection of methicillin resistant *S*.*aureus* (MRSA) was carried out using cefoxitin disk. A standardized bacteriological procedure was followed to maintain correct laboratory results. At regular intervals and whenever a new batch of strain or reagent is prepared, standard strains of *S*.*aureus* ATCC25923, *S*.*pneumoniae*ATCC49619 and *H*. *influenzae* ATCC 49241 were used as positive controls. The sterility of the media was checked by incubating the media overnight before its use.

### Data analysis

Data were entered and analyzed using Statistical Package for Social Science 22 (IBM Corp- Released 2011. IBM SPSS statistics. Armonk, NY: IBM Corp). Descriptive statistics were used to describe relevant variables. Chi-square test and Fishers exact test was obtained to determine association between dependent and independent variables. P-value of < 0.05 was considered statistical significant.

### Ethical considerations

Ethical clearance was secured from Institutional Review Board of College of Medicine and Health Sciences, Bahir Dar University. Permission letter was obtained from FHRH. Written informed consent was obtained from the parents/guardians of children before proceeding to data collection. Children who were positive for the pathogen were reported to physicians for treatment and any other care. Information obtained in the course of the study was kept confidential.

## Results

### Participants’ characteristics

Table 1 depicts the demographic characteristics of the participants. A total of 300HIV infected children with a response rate of (88%) were enrolled in the study. Of them, 153 (51%) were males and 257 (85.7%) were from urban settings. The age range was 6–16 years (median = 11). Majority (89.3%) of the children were living with their family. Educational levels of the mother revealed that 130 (43.3%) were illiterate and 44 (14.7%) had college and above levels of education. Of the total HIV infected children, 235 (78.3%) attended schools (Table 1).

**Table 1:** Demographic characteristics of HIV infected children attending FHRH, 2017 (n = 300).

Demographic variables		Number	Percent
Sex			
	Male	153	51
	Female	147	49
Age (years)			
	6–9	107	35.7
	10–15	193	64.3
Residence			
	Urban	257	85.7
	Rural	43	14.3
Mother’s education			
	Illiterate	130	43.3
	Elementary completed	71	23.7
	Highschool completed	55	18.3
	College and above	44	14.7
Father’s education			
	Illiterate	87	29
	Elementary completed	100	33.3
	Highschool completed	61	20.7
	College and above	52	17.3
Living condition			
	Within the family	268	89.3
	Orphan	32	10.7

### Rate of pharyngeal colonization and frequency of bacterial isolates

Overall, 153 (51%) of children carried pathogenic bacteria in their pharynx. A total of 56.3% aerobic pharyngeal bacteria were isolated from HIV infected children. The most frequent isolate was *S*.*aureus* (29.3%) with colonization rate of *S*.*pneumoniae* (10.3%). Moreover, the colonization rate of *M*.*catarrhalis* and *H*. *influenzae* were 12.3% and 2.3%, respectively (Table 2). Concurrent *S*.*aureus—M*. *catarrhalis* and *S*.*aureus- S*.*pneumoniae* colonization were found in 4.7% and 3.1% of children, respectively (Table 2).

**Table 2:** Pharyngeal carriage and frequency of individual bacterial isolates among HIV infected children attending ART clinic of FHRH, 2017 (n = 300).

Bacterial species	Rate of colonization N (%)
*Staphylococcus aureus*	61 (20.3)
*Morraxella catarrrhalis*	23 (7.7)
*Streptococcus pyogenes*	3 (1.0)
*Streptococcus pneumoniae*	21 (7)
*Haemophilus influenzae*	7 (2.3)
*S*.*aureus + M*.*catarrhalis*	14 (4.7)
*S*.*aureus + S*.*pneumoniae*	10 (3.1)
*S*.*aureus + S*.*pyogen*	3 (2.0)
Total carriage	153 (51%)
Total *S*.*aureus*	88 (29.3)
Total *M*. *catarrrhalis*	37 (12.3)
Total *S*. *pneumoniae*	31 (10.3)
Total *S*. *pyogen*	6 (2)
**Total isolates**	**169 (56.3)**

The overall colonization rate was higher in children from mothers who attained college and above levels of education (79.5%) than illiterates (43.1%) (P = 0.04). The rate of pharyngeal colonization was higher among orphans (56.3%) than other children (50.4%). However, the difference was not statistical significant (P = 0.16) (Table 3). Colonization rate of pharyngeal bacterial pathogens was significantly higher in children without the sign of mal-nutrition (58.5%) compared to others (23.4%)(P = 0.004) (Table 3).

**Table 3:** Association between clinical, other explanatory variables and pharyngeal colonization of bacterial pathogens in HIV infected children attending FHRH, 2017 (n = 300).

Variables	Overall colonization	P-value	Overall colonization	P-value	Overall colonization	P-value	Overall colonization	P-value
	Positive N (%)		Positiv N (%)		Positive N (%)		Positive N (%)	
Sex								
Male (n = 153)	71 (46.4)		54 (36.7)	0.87	9 (8.4)		15 (9.8)	0.7
Female (n = 147)	82 (55.7)	0.37	34 (22.2)		28 (14.5)	0.37	16 (10.9)	
Age (years)								
6–9 (n = 107)	51 (47.7)		21 (19.6)		11 (33.6)		6 (5.6)	
10–15 (n = 193)	102 (52.8)	0.46	67 (34.7)	0.03	26 (37.8)	0.38	25 (13)	0.21
Residence								
Urban (n = 257)	135 (52.5)	0.52	72 (28)		35 (13.7)	0.042	24 (9.3)	
Rural (n = 43)	18 (41.9)		16 (37.2)	0.35	2 (4.5)		7 (16.3)	0.49
Mother’s education								
Illiterate (n = 130)	56 (43.1)		41 (31.5)		10 (7.7)		15 (11.5)	
Elementary completed (n = 71)	29 (40.8)		13 (18.3)		6 (8.3)		7 (9.9)	0.92
Highschool completed (n = 55)	33 (60)	0.04	19 (34.5)	0.24	10 (18.2)		6(10.9)	
College and above (n = 44)	35 (79.5)		15 (34.1)		11 (25.6)	0.002	3 (6.8)	
Father’s education								
Illiterate (n = 87)	42 (48.3)		29 (33.3)	0.74	3 (3.4)		13(14.9)	0.29
Elementary completed (n = 100)	45(45)		29 (29)		15 (15)		5 (5)	
Highschool completed (n = 61)	33 (54.1)	0.59	15 (24.6)		9 (14.8)		8(13.1)	
College and above (n = 52)	33 (53.2)		15 (28.8)		10 (23.8)	0.59	5(11.4)	
Living condition								
Within the family (n = 268)	135 (50.4)		81 (30.2)	0.33	31 (11.7)		26 (9.7)	
Orphan (n = 32)	18 (56.3)	0.16	7 (21.9)		6 (28.6)	0.16	5 (15.6)	0.46
Presence of younger siblings								
Yes (n = 82)	28 (34.1)	0.78	28 (34.1)	0.36	12 (14.6)	0.9	7 (8.5)	0.79
No (n = 218)	141 (64.7)		60 (27.5)		25 (11.5)		24 (11)	
Exposure to passive smoking in the house								
No (n = 276)	147 (53.3)	0.09	84 (30.4)	0.39	37	0.13	29(10.5)	1
Yes (n = 24)	6 (25)		4 (16.7)		0		2(8.3)	
Attending school								
Yes (n = 235)	128 (54.5)	0.15	76 (32.2)	0.045	29 (12.3)	0.99	26(11.1)	0.38
No (n = 65)	25 (38.5)		12 (18.5)		8 (12.3)		3(4.6)	
Living with health care workers in the house								
Yes (n = 37)	24 (64.9)	0.45	9(24.3)		3 (8.1)	0.54	6(16.2)	0.25
No (n = 263)	129 (49)		79 (30)	0.53	34 (12.9)		25(9.5)	
Respiratory tract infection for the last month								
Yes (n = 25)	15 (60)	0.74	4 (16)		4 (8)	0.44	2 (8)	
No (n = 275)	138 (50.2)		84 (30.5)	0.78	33 (12)		29 (10.5)	1
Sign of malnutrition								
Yes (n = 64)	15 (23.4)	0.004	14 (21.9)		8 (12.5)	0.42	3 (4.7)	
No (n = 236)	138 (58.5)		66 (28)	0.44	29 (12.3)		28 (11.9)	0.13
Running nose for the last two weeks								
Yes (n = 60)	36 (60)	0.44	24 (40)		8 (13.3)	1	6 (10.1)	
No (n = 240)	117 (48.8)		64 (26.7)	0.07	29 (12.1)		25 (10.4)	0.89
Nail priming with teeth								
Yes (n = 57)	42 (73.7)	0.06	13 (22.8)	0.75	13 (22.8)	0.59	8 (14)	0.42
No (n = 243)	111 (45.7)		75 (30.9)		24 (9.9)		23 (9.5)	
Coryza for the last two months								
Yes (n = 38)	22(57.9)	0.73	11(28.9)	0.88	0	0.048	13 (15.8)	0.25
No (n = 262)	131 (50)		77 (29.4)		37(14.1)		25 (9.5)	
Habit of nose picking								
Yes (n = 48)	28 (58.3)	0.63	16(33.3)	0.85	8(16.7)	1	6 (12.5)	0.58
No (n = 252)	125(49.6)		72 (28.6)		29 (11.5)		25 (9.9)	

The proportion of *S*.*aureus* colonization was higher in older (37.6%) compared to younger (22.4%) children (P = 0.03). It was higher in children who had coryza (40%) compared to those who had not (26.7%) (P = 0.046). The colonization rate of *S*.*aureus* was higher among school (35.1%) compared to non- school attending children (19.5%) (P = 0.045) (Table 3). Colonization rate of *M*.*catarrhalis* was higher in children from mothers with education college and above (25.6%) than illiterates (7.7%) (P = 0.002). Moreover, colonization rate of *M*.*catarrhalis* was higher in urban (13.7%) compared to rural (4.5%) residents (P = 0.042) (Table 3).

### Drug resistance profiles of the bacterial isolates

Majority of the bacterial isolates were resistant to penicillin (68.5%) and cotrimoxazole (52.5%). *S*.*aureus* isolates were resistant to penicillin (84%) and cotrimoxazole (51.1%). The proportion of MRSA was 29 (33%). However, *S*.*aureus* isolates were susceptible to chloramphenicol (6.8%), clindamycin (26.1%), ciprofloxacin (27.3%) and erythromycin (35.2%). *M*.*catarrrhalis* isolates were resistant to penicillin (94.6%), erythromycin (86.5%), cotrimoxazole (78.4%) and clindamycin (73%).*S*.*pneumoniae* isolates revealed 26.7%, 35.5% and 36.7% resistance to cotrimoxazole, erythromycin, tetracycline and chloramphenicol, respectively (Table 4).

**Table 4:** Antimicrobial resistance profiles of respiratory bacteria among HIV infected children attending at FHRH, 2017.

Antimicrobials	*S*. *aureus*		*S*. *pyogenes*		*S*. *pneumoniae*		*M*. *catarrrhalis*		*H*.*influenzae*		Total	
	# T	R%	# T	R%	# T	R%	# T	R%	# T	R%	# T	R%
Cefoxitin	88	29 (33)	NA	NA	NA	NA	NA	NA	NA	NA	88	29 (33)
Clindamycin	88	23 (26.1)	6	0	NA	NA	37	27 (73)	NA	NA	131	50 (38.2)
Cotrimoxazole	88	45 (51.1)	NA	NA	31	8 (26.7)	37	29 (78.4)	6	3 (50)	162	85 (52.5)
Ciprofloxacin	88	24 (27.3)	6	1 (20)	NA		37	6 (16.2)	6	1 (16.7)	137	32 (23.4)
Erythromycin	88	31 (7.4)	6	1 (20)	31	11 (35.5)	37	32 (86.5)	NA	NA	162	75 (46.3)
Tetracycline	88	19(21.6)	NA		31	11 (35.5)	37	14 (37.8)	6	3 (50)	162	47 (29)
Penicillin	88	74 (84.1)	6	0	31	6 (19.4)	37	35 (94.6)	6	0	168	115 (68.5)
Chloramphenicol	88	6 (6.8)	6	3 (50)	31	12 (38.7)	37	6 (16.2)	6	4 (66.7)	168	31 (18.5)
**Total**	**157**	**251 (35.6)**	**30**	**5 (16.7)**	**155**	**55 (35.5)**	**259**	**149 (57.5)**	**30**	**11 (36.7)**	**1178**	**464(39.4)**

# T: Number of isolates tested, R%: percentage of resistant isolates, NA: Not applicable

### Multi-drug resistance profiles of the isolates

Thirty one (18.6%) of the isolates was susceptible to all drugs tested. One hundred thirty two (79%) of the isolates were resistant to one and more drugs tested. MDR to two and more different classes of drugs were found in 99 (59.3%) of the isolates. The overall MDR rate among *S*.*aureus* and *M*.*catarrhalis* isolates were 65.9% and 78.4%, respectively (Table 5).

**Table 5:** Antibiogram of respiratory bacteria isolated from HIV infected children attending ART clinics of FHRH, 2017.

Bacterial species	Ro	R1	R2	R3	R4	R5	R6	Overall, MDR
*M*. *catarrrhalis* (n = 37)	5 (18.9)	3 (10.8)	7 (13.5)	7 (18.9)	5 (13.5)	6 (16.2)	4 (10.8)	29(78.4)
*S*.*pneumoniae*(n = 31)	15 (36.7)	8 (6.7)	5 (13.5)	2 (21.6)	0	0	0	7 (22.6)
*S*.*aureus* (n = 88)	8 (11.1)	22 (22.2)	35 (44.4)	11 (11.1)	12 (11.1)			58 (65.9)
*H*. *catarrrhalis* (n = 6)	1 (33.3)	1(16.7)	1(16.7)	2 (33.3)				3(50)
*S*. *pyogens* (n = 5)	2 (40)	1 (20)	2 (40)	0	0	0	0	2 (40)
**Total (n = 167)**	**31 (18.6)**	**35 (21)**	**50 (29.9)**	**20 (12)**	**17 (10.2)**	**6 (36)**	**4 (2.4)**	**99 (59.3)**

R0: susceptible to all antimicrobials tested; R1, R2, R3, R4, R5, R6: Resistance to one, two, three, four, five and six antimicrobials, respectively

## Discussion

Although cotrimoxazole prophylaxis and pneumococcal conjugate vaccine for HIV infected individuals have been started to prevent the development of some of the most serious and common childhood bacterial opportunistic infections, the current pharyngeal colonization rate of bacterial pathogens was in accord with various studies for individual pathogens [23–27]. These imply that HIV infected children could be sources of bacterial infection to other school children and families posing high risk of developing invasive and non-invasive bacterial diseases. Five different bacterial species were isolated from HIV infected children. *S*.*aureus* was the predominant organism carried by the HIV positive children in this study. However, in similar studies at Cambodia [26] and Turkey [27], *M*. *catarrrhalis* was reported as the leading isolate. These reflect the presence of variations in respiratory bacteria composition which might be due to difference in geographical areas and other factors [1, 4, 6].

In this study, the proportion of bacterial pharyngeal colonization was significantly higher in HIV infected children from mothers with educational attainment college levels and above than others (P = 0.04). This might be due to acquisition of the organisms from the school. Moreover, bacterial pharyngeal colonization rate was significantly higher among children who had no sign of malnutrition compared to children with the sign of malnutrition (P = 0.004) which might be associated with acquiring the bacteria from the school or day- care center. The above explanation is also supported by Table 3 hence increased age, schooling and living in urban area were significantly associated for *S*.*aureus* and *M*.*catarrhalis* colonization, respectively.

The pharyngeal colonization rate of *S*.*pneumoniae* (10.3%) in the present study is comparable to earlier findings in Cameroon (11.6–18.2%) [20], Seoul, Korea (12.2%) [3] and Thailand (12.9%) [24]. However, higher colonization rate was documented in North and South Ethiopia (41–43.8%) [9, 25], Estonia (17%) [28], Uganda (18%) [29], West Bengal (15.6%) [30], Ghana (30.5%) [31], Rural Uganda (58.6%) [32], Tanzania (56%) [33]and South Africa (22.2%) [34]. On the other hand, lower (5.1%) colonization rate was documented in Cambodia [26]. The variation in the colonization rate might be due to difference in the age range of participants, vaccination status, living condition, season of data collection and methodology. Moreover, the present study performed pharyngeal swabs while others performed either nasopharyngeal or oropharyngeal swabs [1, 4].

In this study, the pharyngeal colonization rate of *S*.*aureus* (29.3%) is consistent with an earlier finding in Mekelle, Ethiopia (32.5%) [23]. However, it was lower than studies done in Gondar, Ethiopia (51.5%) [9] and Tanzania (66%) [33]. On the other hand, it was higher than previous reports from Cameroon (6–12.3%) [20], Ghana (22%) [31], South Africa (20.4%) [34] Seoul, Korea (18.2%) [3] and south central Tanzania (23.2%) [35]. The variation in colonization rate might be associated with variation in HIV status of children [6], type of specimen collected and age of the participants.

The carriage rate of MRSA in the present study (33%) was higher than previous studies in Mekelle, Ethiopia (2.4%) [23] and Ghana (3.4%) [3]. The higher carriage rate of MRSA in the current study might be due to the high visit of these patients to the health care settings, since repeated visits to health care settings or contact with the hands of health care workers in HIV infected individuals is the major risk factor for the colonization.

The proportion of pharyngeal colonization of *M*.*catarrrhalis* (12.3%) in the current study was higher than previous reports in Cambodia (6.2%) and Kyrgz (7.2%) [26]. However, it was lower than findings reported in Estonia (16%) [28], Ghana (39.8%) [31], Tanzania (50%) [33], south central Tanzania (90.8%) [35], Seoul, Korea (20.2%) [3] and Turkey (23.9%) [27]. The variation might be related with differences in age of the participant and type of samples collected. However, due to paucity of previous data in Ethiopia, comparison was not made.

In this study, the colonization rate of *H*.*influenzae* (2%) from the pharynx of HIV infected children was comparable with a previous report in Dhaka city (1.5%) [36]. However, it was lower than findings reported in Tanzania (14%) [33], West Bengal (13.8%) [30], Botswana (3–8%) [37], South Africa (21.8%) [34], Cameron (13.8–24.4%) [20], Seoul, Korea (18.9%) [3], Estonia (16%) [28] and Turkey (7.2%) [27]. The lower colonization rate of *H*.*influenzae* in the present study might be associated with the variation in age range of the study participants as under five children were not included in the present study.

In this study, majority of the isolates were resistant to cotrimoxazole. This was in agreement with previous studies in Dares-Salaam, Tanzania [38] and Uganda [29]. The high level of resistance could be due to the fact that cotrimoxazole is the least expensive orally administered antibiotic and is easily available over the counter in many settings and the drug has been widely used for the prophylaxis of opportunistic infections in HIV infected patients.

In the present study, most (84.5%) of *S*.*aureus* isolates were resistant to penicillin. This was consistent with previous findings reported in Northwest Ethiopia [12] and Ghana [31] where 90–100% resistance has been reported. It is a fact that most strains of *S*.*aureus* are currently resistant to penicillin through beta—lactamase production and alteration of the penicillin binding proteins.

Though the level of *S*.*pneumoniae* resistance to cotrimoxazole (22.6%) observed in the present study is lower than previous findings in Gondar, Ethiopia (29.2%) [12], Gambian village (39%) [39], South west Ethiopia (43.7%) [23], rural Uganda (58.3%) [32], Uganda (98.9%) [29], Dares-Salaam, Tanzania (82.6%) [38], Kuwait (62.8%) [40], Indonesia (59%) [41], Estonia (62.1%) [28], India (91%) [42] and Thailand (78.9%) [24], the current resistance level suggests the spreading of drug resistant *S*. *pneumoniae* in the community which might be due to the use of cotrimoxazole as a prophylaxis in HIV infected patients. Moreover, cotrimoxazole is cheap and readily available to the community that might leads to frequent use of it.

Relatively higher level of *S*.*pneumoniae* resistance to erythromycin (35.5%), tetracycline (35.5%) and chloramphenicol (38.7%) were reported in the present study. These were concurrent with findings documented in Gondar, Ethiopia [12] and Gambia [43]. This might be associated with easily availability of these drugs, self-medication, illegal purchase without medication, wide spread of these drugs for treatment of various infections in the setting which leads to misuse of antibiotics.

In this study, the overall MDR rate of *S*.*pneumoniae* was 22.6% [Table 5]. This was higher than studies in Ghana (16.7%) [31], Italian city (16.5%) [44], India (15.9%) [42], Dares-Salaam Tanzania (16.5%) [38], South west Ethiopia (17.7%) [23] and Thailand (31.6%) [24]. In this study most (94.7%) of *M*.*catarrrhalis* isolates were resistant to penicillin. This was comparable to previous findings in Cameron [20] and Turkey [30] where100% and 84% level of resistance were reported, respectively. This might be associated with inactivation of the drug by beta-lactamase enzymes as many strains of *M*.*catarrhalis* are beta-lactamase producers. The relatively higher level of MDR *S*.*pneumoniae* alarms the treatment of infections caused by *S*.*pneumoniae* to be guided by culture and antimicrobial susceptibility testing.

The overall MDR rate of *M*.*catarrrhalis* in this study was 78.4% (Table 5). This was similar with previous findings in Ghana (57.4%) [31]. This is a really shocking level of resistance from medically important commensal bacteria that would be difficult to manage if it brings opportunistic infections.

The majority of *M*. *catarhalis* isolates in this study were resistant to erythromycin, cotrimoxazole and clindamycin. However, comparison was not made due to the lack of documented data. The high level of *M*.*catarhalis* to different classes of antibiotics might be associated with the ability of the pathogen to have multiple mechanism of resistance and wide spread of the drugs favors selective pressure on normal colonizers.

Because of cross-sectional nature of the study, the determinant factors of colonization could not be obtained. The different serotypes of each species were not characterized due to limited laboratory infrastructure.

## Conclusions

Pharyngeal colonization of respiratory bacteria in HIV infected children is a major public health problem. Majority of the isolates were resistant to penicillin and cotrimoxazole. Multidrug resistant *S*.*aureus*, *M*.*catarhalis* and *S*.*pneumoniae* were alarmingly high. Regular screening of HIV infected children is recommended for the follow-up of these children.

## Supporting information

S1 Table Questionnaire for collection of socio-demographic characteristics, clinical and other explanatory variables of HIV infected children attending at Felegehiwot Referral Hospital. (DOCX)
