# Supplementary material for: Pharyngeal colonization and drug resistance profiles of Morraxella catarrrhalis, Streptococcus pneumoniae, Staphylococcus aureus, and Haemophilus influenzae among HIV infected children attending ART Clinic of Felegehiwot Referral Hospital, Ethiopia
Source: PLoS One. 2018 May 10;13(5):e0196722. doi: 10.1371/journal.pone.0196722 (PMC5944927; doi:10.1371/journal.pone.0196722)
Supplement: S1 Table — (DOCX) [file pone.0196722.s001.docx]

ኤች.አይቪ በደማቸው ከሚገኝባቸው ህጻናት የላይኛው የመተንፈሻ አካል ለይ በሽታ አምጭ ባክቴሪያ የመሸከም ስርጭት ለማዎቅ የተዘጋጅ መጠይቅ ( In Original language (Amharic))

1. ጾታ ወንድ ሴት

2. ዕድሜ (በዓመት) 6-9 10-15

3. የመኖሪያ አድራሻ ከተማ ገጠር

4. የእናት የትምህርት ደረጃ ያልተማረች አንደኛ ደረጃ ያጠናቀቀች ሁለተኛ ደረጃ ያጠናቀቀች ኮሌጅ እና ከዛ በላይ

5. የአባት የትምህርት ደረጃ ያልተማረ አንደኛ ደረጃ ያጠናቀቀ ሁለተኛ ደረጃ ያጠናቀቀ ኮሌጅ እና ከዛ በላይ

6. ህጻኑ/ኗ ከማን ጋር ነው የሚኖረው/የምተኖረው ከቤተሰብ ጋር ወላጅ አልባ

7. በቤት ውስጥ ከህጻኑ/ኗ በዕድሜ የሚያንሱ ህጻናት አሉ አዎ የለም

8. በቤት ውስጥ ሲጋራ የሚያጨስ አለ አዎ የለም

9. ህጻኑ/ኗ ትምህርት ይማራል/ትማራለች አዎ አይማርም/አትማርም

10. በቤት ውስጥ የሚኖር የጤና ባለሙያ አለ አዎ የለም

11. ህጻኑ/ኗ በባለፈው አንድ ወር ውስጥ የመተንፈሻ አካላት ታሞ/ማ ነበር አዎ የለም

12. ህጻኑ/ኗ የምግብ እጥረት ምልክት አለበት/አለባት አዎ የለም

13. ህጻኑ/ኗ በባለፈው አንድ ሳምንት ውስጥ ከአፍንጫ የሚወጣ ተደጋጋሜ የሆነ ፈሳሽ ነበረው/ነበራት

አዎ የለም

14. ህጻኑ/ኗ ጥፍሩን/ሯን በጥርስ ያሳጥራል/ታሳጥራለች አዎ የለም

15. ህጻኑ/ኗ በባለፈው ሁለት ወር ውስጥ ተደጋጋሚ የሆነ የአፍንጫ መቆጣት ነበረው/ራት

አዎ የለም

16. ህጻኑ/ኗ አፍንጫውን/ዋን የመነካካት ልምድ አለው/አላት አዎ የለም

Questionnaire for collection of socio-demographic characteristics, clinical and other explanatory variables of HIV infected children attending at Felegehiwot referral Hospital

| 1. **Back ground variables** | **Responses** |
| --- | --- |
| 1. Age |  |
| 1. Sex | 1. Male 2. Female |
| 1. Living condition | 1. Within a family 2. Orphan |
| 1. Fathers education | 1. Illiterate 2. Elementary completed 3. Highschool completed 4. College and above |
| 1. Mother’s education | 1. Illiterate 2. Elementary completed 3. Highschool completed 4. College and above |
| 1. Residence | 1. Urban 2. Rural |
| 1. **Other explanatory factors** |  |
| 1. Have you been exposed to passive smoking in the house | 1. Yes 2. No |
| 1. Are you attending school | 1. Yes 2. No |
| 1. Attendance at day care center (participant or sibling) | 1. Yes 2. No |
| 1. Is there health care provider at home | 1. Yes 2. No |
| 1. Presence of younger sibling | 1. Yes 2. No |
| 1. **Clinical related factors** |  |
| 1. Respiratory infection in the last month | 1. Yes 2. No |
| 1. Signs of malnutrition | 1. Yes 2. No |
| 1. Runny/stuffy nose in the last 2 weeks | 1. Yes 2. No |
| 1. Cough/expectoration in the last 2 weeks | 1. Yes 2. No |
| 1. Coryza/sneezing in the last 2 weeks | 1. Yes 2. No |
| 1. Habit of nose picking | 1. Yes 2. No |
| 1. Habit of nail priming with teeth | 1. Yes 2. No |
